# Fast and Flexible Multi-Step Cloth Manipulation Planning Using an Encode-Manipulate-Decode Network (EM*D Net)

**DOI:** 10.3389/fnbot.2019.00022

**Published:** 2019-05-31

**Authors:** Solvi Arnold, Kimitoshi Yamazaki

**Affiliations:** Department of Mechanical Systems Engineering, Shinshu University, Nagano, Japan

**Keywords:** planning, neural networks, forward models, deformable objects, manipulation, machine learning

## Abstract

We propose a deep neural network architecture, the Encode-Manipulate-Decode (EM*D) net, for rapid manipulation planning on deformable objects. We demonstrate its effectiveness on simulated cloth. The net consists of 3D convolutional encoder and decoder modules that map cloth states to and from latent space, with a “manipulation module” in between that learns a forward model of the cloth's dynamics w.r.t. the manipulation repertoire, in latent space. The manipulation module's architecture is specialized for its role as a forward model, iteratively modifying a state representation by means of residual connections and repeated input at every layer. We train the network to predict the post-manipulation cloth state from a pre-manipulation cloth state and a manipulation input. By training the network end-to-end, we force the encoder and decoder modules to learn a latent state representation that facilitates modification by the manipulation module. We show that the network can achieve good generalization from a training dataset of 6,000 manipulation examples. Comparative experiments without the architectural specializations of the manipulation module show reduced performance, confirming the benefits of our architecture. Manipulation plans are generated by performing error back-propagation w.r.t. the manipulation inputs. Recurrent use of the manipulation network during planning allows for generation of multi-step plans. We show results for plans of up to three manipulations, demonstrating generally good approximation of the goal state. Plan generation takes <2.5 s for a three-step plan and is found to be robust to cloth self-occlusion, supporting the approach' viability for practical application.

## Introduction

Within the area of robotic manipulation planning, deformable objects pose a particularly tough challenge. Manipulation changes the shape of such objects, so the common strategy of acquiring a 3D model of the object and planning w.r.t. this model is of little use. Even just predicting the object shape that will result from a given manipulation is far from trivial. Maybe the most common type of deformable object manipulation is cloth manipulation. Cloth exemplifies the difficulties stated above, yet humans manipulate cloth routinely and with ease, without much thought. We seem to acquire an intuitive sense of how cloth reacts to our manipulations. Replicating this ability in AI is a challenge of both theoretical and practical interest.

One point of particular theoretical interest is that our affinity with cloth goes beyond fixed goal-based routines, yet does not fit rule-based reasoning patterns. Despite being notoriously hard to formalize (or even verbalize), our affinity with cloth generalizes well to novel situations. This puts cloth manipulation into somewhat of a gray zone, that present day AI does not yet have a clear solution for.

Practical interest in cloth manipulation derives from the fact that cloth is ubiquitous in our everyday lives. Many everyday chores involve cloth manipulation in one form or another, so efficient cloth manipulation capabilities would be an important feature in household support robots.

### Related Work in Cloth Manipulation

Much work in multi-step cloth manipulation avoids the need to plan by assuming fixed, hand-designed manipulation procedures. Such procedures can be quite effective for specific tasks (Maitin-Shepard et al., 2010; Koishihara et al., 2017; Yuba et al., 2017). Assuming a circumscribed starting situation and fixed outcome allows comparatively quick operation with limited computational cost, making this type of approach feasible for real-world applications where the same tasks have to be performed over and over, such as in industrial settings. However, there is no flexibility to accommodate new goals; every new goal state requires a new, human-provided, plan.

To flexibly realize variable goals requires an ability to plan ahead, to foresee the outcomes of individual actions and string actions together accordingly. This naturally leads to simulation-based approaches. While simulation provides high flexibility in terms of the manipulations that can be considered (Kita et al., 2014; Li et al., 2015), application in planning faces at least two major hurdles. The first is computational cost. Simulating a single manipulation is computationally expensive, and planning a sequence of manipulations generally requires consideration of a substantial number of possibilities. This in turn makes explicit search for manipulation sequences slow and impractical. The second hurdle is that obtaining an accurate deformation model of a given object is a difficult problem in itself, an issue that gets more pressing as more complex manipulations and longer sequences of manipulations are considered.

A promising intermediary approach works by retrieving and modifying manipulations from a database (Lee et al., 2015). This offers more operational flexibility than fixed procedures at a smaller computational cost than the simulation-based approach. However, present demonstrations of this approach still assume a fixed goal, and whereas the cost of database retrieval and deformation operations is less than full-fledged simulation, it is not clear whether this approach can be made efficient enough to perform free-form planning in real-time.

Recent years have seen increasing interest in the use of neural networks for manipulation problems. Impressive results have been demonstrated in grasp point detection for rigid objects (Lenz et al., 2015), and visuomotor policy learning (Levine et al., 2016). Given neural networks' natural affinity for fuzzy subject matter, they may have the potential to bring major progress to deformable object manipulation. Neural network-driven grasp point detection has been applied in a bed-making task (Seita et al., 2018). Interesting results have been reported on a dual neural network approach to cloth folding, combining a convolutional autoencoder and a time-delay neural network to achieve fine control over manipulation motions (Yang et al., 2017). Whereas the goal is fixed, motion is guided by network-generated predictions of the very near future, thus realizing some degree of foresight. Neural network-driven prediction has also been employed for prediction of forces exerted on human subjects in a dressing task (Erickson et al., 2018).

Despite these advances, open-goal, multi-step manipulation planning for deformable objects remains largely unexplored territory.

### Related Work in Model-Based Learning

There is increasing evidence from neuroscience that humans learn, in part, by acquiring forward models (Gläscher et al., 2010; Liljeholm et al., 2013; Lee et al., 2014). Human ability to generalize implicit knowledge of cloth dynamics to novel circumstances suggests that we acquire forward models of these dynamics. Forward models are commonly used in model-based control and planning, but in the case of cloth manipulation planning, the use of explicit forward models (i.e., physical simulation) is problematic due to computational cost and the difficulty of obtaining an accurate model, as discussed above. However, it has been demonstrated that neural networks can be trained as forward models. Of particular relevance here is (Wahlström et al., 2015) for the use of a neural network trained as a forward model in latent space. The proposed model takes high-dimensional observations (images) of a low-dimensional control task as inputs, maps these observations into low-dimensional latent representations (by means of PCA followed by an encoder network), feeds these through a network functioning as a forward model, and then maps the outputs of this network to high-dimensional predictions of future states. This model is then used to search for control signals that bring about a fixed goal.

Also related is (Watter et al., 2015). Here too, an encoder network is used to map high-dimensional observations to low-dimensional latent representations. The forward model takes the form of linear transformations in latent space (although a non-linear variant is considered as well). We return to these and other related neural network studies in the discussion section. In the context of cloth manipulation, use of a neural network as forward model allows us to side-step the computational cost of explicit simulation (replacing it with forward propagation through the network), as well as the burden of acquiring an accurate model of a given cloth item (instead, the forward model is learned from data).

### Contributions and Limitations

This paper presents a fully connectionist approach for efficient deformable object manipulation planning, based on forward modeling of the object's deformation dynamics with respect to a given manipulation repertoire. We avoid explicit simulation and database matching/retrieval, yet realize a substantial degree of flexibility along with fast operation time. Core of the system is a modular neural network architecture, composed of 3D convolutional encoder and decoder modules and a fully connected manipulation module. Given a start and goal state, the network is used to search for the manipulation sequence that produces the latter from the former, by means of error back-propagation w.r.t. the manipulation input. We can search for manipulation sequences of various lengths by varying the number of recurrent propagation loops through the manipulation module. In the present paper, we apply this manipulation planning approach to free-form manipulation on a (simulated) square cloth.

The main contributions of this work are as follows.

- We propose a neural network architecture for associating manipulations with changes in cloth states, trainable on individual manipulation examples from a comparatively small dataset.
- We show that this network can predict cloth manipulation outcomes.
- We show that this network can be used to generate single and multi-step manipulation plans in seconds, by means of back-propagation w.r.t. the manipulation inputs.

An important distinction between our system and most existing work in cloth manipulation is that our system is, to a large degree, task-agnostic. The task domain and manipulation repertoire are determined by the dataset the system is trained on. We believe this should provide a high degree of flexibility for application to various task domains and manipulation repertoires.

This has consequences for system evaluation as well. The free-form manipulation task we adopt here for evaluation is not intended to represent or resemble any particular practical cloth manipulation task, nor was the manipulation repertoire designed with any specific robotic platform in mind. Instead, our experimental setup is designed to assess the system's capabilities on a broad domain with a basic manipulation repertoire that could be realized on a wide variety of robotic platforms. The motivation for this choice is two-fold.

The first motivation is that success on a broad task would suggest that the approach is viable for a broad variety of more specific tasks. For use in a practical, constrained application, one would want to use a dataset that covers a domain suited to the application, with a manipulation repertoire suited to the specific robotic platform under consideration.

The second motivation is the long-term goal of pursuing a generalized affinity with cloth objects. Human affinity with cloth goes far beyond folding towels and clothes into neat rectangles. We quickly drape a dish towel over the back of a chair when a goal more urgent than drying the dishes presents itself. We extract sizable bed sheets from a washing machine without them sweeping over the floor with little effort. We intuit what will or will not fit into a coat pocket. Much of human cloth manipulation seems better characterized as the flexible application of a general understanding of cloth dynamics than as mastery of a large collection of individual micro-tasks. Progress toward broad generalized cloth manipulation abilities for robots requires that we try and push toward methods that offer increasingly high degrees of generality. In the context of this goal, the value of our results lies not in their practical applicability, but in the fact that they represent progress toward higher generality.

As will be clear from the above, our purpose in this work is not to excel at any one specific example of cloth manipulation, and practical applicability of the system as trained on our dataset is limited at best. Also, whereas we believe that our approach should be viable for a range of task settings more specific than ours, we expect its applicability to highly constrained tasks to be limited: planning ability is only meaningful on tasks that present significant variability and require some level of system autonomy.

## Task Design

The problem of manipulation planning can be formulated as follows: Given a state domain consisting of possible state set *S*, a manipulation (action) domain consisting of possible manipulation set *M*, and states *s*_*a*_ and *s*_*b*_ ϵ *S*, find a manipulation sequence (action sequence) *p*_*ab*_ = <*m*_0_*, …, m*_*n*−1_> with *m*_*i*_ ϵ *M* such that applying manipulation sequence *p*_*ab*_ starting in state *s*_*a*_ will produce state *s*_*b*_.

**Figure 1:**
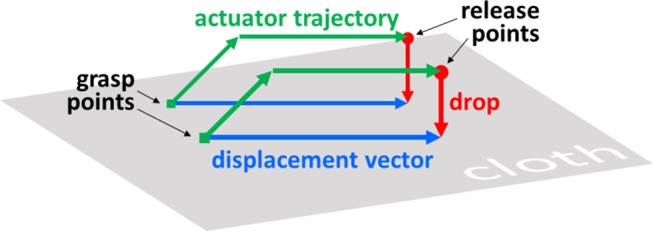
Schematic of cloth manipulation.

In this paper we consider the task of manipulating a square piece of cloth from one configuration into another. We let states represent the cloth in some stable configuration. Manipulations are defined as triplets of real-valued 2D vectors. The first two vectors indicate the x and y coordinates where the cloth is picked up (grasp points below), and the third vector (displacement vector below) indicates the horizontal movement of the grasp points (both points are moved in parallel and by the same distance, so one vector suffices). Figure 1 illustrates how such manipulations are translated into actuator trajectories. Plans are sequences of manipulations. The height to which the grasp points are lifted is fixed.

The manipulation format is intentionally somewhat minimalistic. Every additional dimension also adds to the complexity of the planning process, so any aspects that can be resolved locally are best excluded from the planning process. In simulation, we perform manipulations by fixing the relevant vertices of the cloth mesh to a non-colliding actuator. The complexities of performing cloth manipulations with any given physical actuator are not considered in the present paper. In ongoing work, we are integrating the planning system described here with a physical dual-armed robot platform (Tanaka et al., 2018).

## System Architecture

The system is composed of three modules: encoder module E, manipulation module M, and decoder module D. E maps (encodes) state *s*_*i*_ to its latent representation *c*_*i*_:

(1) $$\begin{array}{r} \begin{array}{c} E\left(s_{i}\right)=\;c_{i}. \end{array} \end{array}$$

M maps latent representation *c*_*i*_ and manipulation *m*_*i*_ to a prediction *ĉ*_*i*+1_ of *c*_*i*+1_, the latent representation of the state *s*_*i*+1_ that results from applying manipulation *m*_*i*_ to *s*_*i*_:

(2) $$\begin{array}{r} \begin{array}{c} M\left(c_{i},m_{i}\right)={\hat{c}}_{i+1}. \end{array} \end{array}$$

D maps (decodes) latent representation *c*_*i*_ (or *ĉ*_*i*_) to an approximation *ŝ*_*i*_ of state *s*_*i*_:

(3) $$\begin{array}{r} \begin{array}{c} D\left(c_{i}\right)={\hat{s}}_{i}. \end{array} \end{array}$$

Given a state *s*_*i*_ and a manipulation *m*_*i*_, *D(M(E(s*_*i*_*),m*_*i*_*))* computes a prediction of the outcome *s*_*i*+1_. Mapping to latent space before applying M, and mapping back to regular space afterwards, serves two purposes. The first is dimensionality reduction. States in our task are 16384D. Applying manipulations directly on states of this dimensionality is computationally costly and hard (if not impossible) to train. Modules E and D map states to more manageable 512D latent representations. The second reason is manipulability. Depending on how a state is represented, it may be easier or harder to apply specific manipulations to it. By training E, M, and D in compound fashion, the E and D modules are forced to learn a latent representation format that makes M's life easy, i.e., lends itself well to application of the manipulation repertoire.

Movement of one point of a cloth often affects the cloth's shape over a broad region in non-trivial but highly structured ways. For predicting these effects, the substrate of a voxel representation is likely far from ideal. We let E map voxel representations to latent representations with no imposed spatial structure, so the learning process is free to find a way of representing the cloth's spatial contingencies that facilitates prediction of manipulation outcomes.

**Figure 2:**
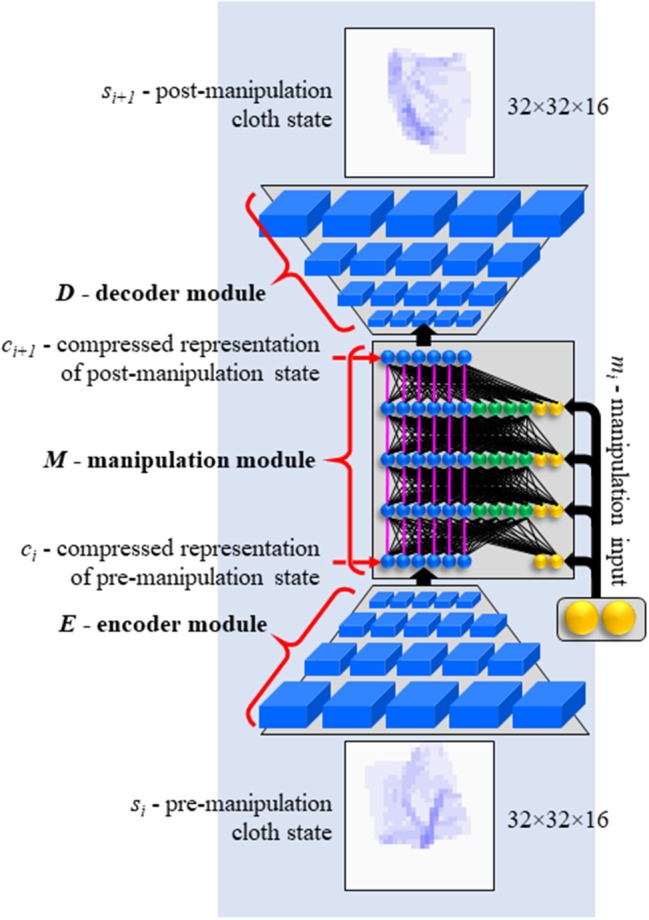
Concept image of EMD network. Functionally denoted as *D(M(E(s*_*i*_*),m*_*i*_*))*. See Table 1 for actual sizes.

Each module is instantiated as one neural network. Encoder E and decoder D are structured like the bottom and top halves of a 3D convolutional autoencoder. Manipulation module M is a modified multi-layer perceptron. A concept image of the network is shown in Figure 2. The network is implemented in TensorFlow (Abadi et al., 2015). Network specifications are given in Table 1.

**Table 1:** Network architecture specifications.

**3D CONVOLUTIONAL ENCODER (E)/UP-CONVOLUTIONAL DECODER (D)**	
Layers	6
Feature maps / layer	1 (input), 32, 32, 64, 128, 256, 512 (output). Order reversed in decoder.
Kernel size	3 × 3 × 3 (all layers)
Strides	2 × 2 × 1 on the first layer (E) / last layer (D) 2 × 2 × 2 on all other layers
Activation function	tanh
**MANIPULATION MODULE (M)**	
Layers	10 (5 in configuration C2)
Input layer size	512+6
Hidden layer size	512+512+6 (512+512 in configuration C0)
Output layer size	512
Activation function	tanh

We avoid pooling, because it discards important spatial information. The use of pooling between convolution layers is usually motivated by the partial translation invariance and dimensionality-reduction it affords, but in the present system the former is detrimental and the second can as well be obtained with strided convolution, which does not destroy spatial information. As can be inferred from the strides and map counts given above, E maps 32 × 32 × 16 × 1 inputs to 1 × 1 × 1 × 512 outputs (here the first three dimensions are spatial, the fourth is the channel dimension), and D does the inverse. The latent representations only have extension in the channel dimension, meaning they have no imposed spatial structure.

Cloth state input is given in the form of a binary voxel rasterisation of the (simulation-generated) cloth mesh, at the 32 × 32 × 16( × 1) resolution taken by E. Each voxel takes a value of 1 if one or more vertices of the cloth mesh fall in that voxel, and a value of 0 otherwise. Before rasterisation, we multiply the vertices' z coordinates by a factor 4 to emphasize height variations (effectively increasing resolution on the z-axis by a factor 4). This is important to ensure that creases in the cloth (which do not have much height but do provide important shape information) do not get lost in rasterisation.

We scale the view-port of the voxel space so that the cloth, shape-wise, fully fits inside it in any plausible stable shape configuration. However, repeated manipulations can move the cloth by a substantial distance, which would quickly take it out of the range of the viewport. To keep the cloth always fully in view, we introduce periodic boundary conditions on the x and y axes. That is, we bring vertices' x and y coordinates into the [-1, 1] range using *x*′ = (*x* + 1) *mod* 2 − 1 (and same for y).

**Figure 3:**
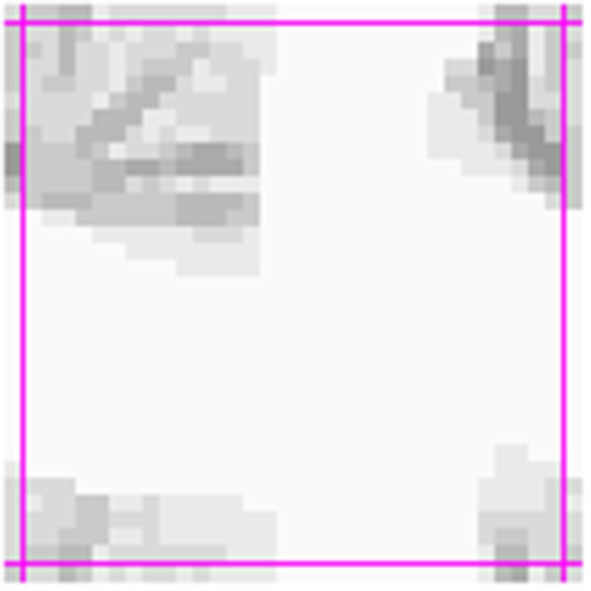
Top-down view (voxel value means over z-axis) of a voxel representation of a state with the cloth wrapped around the periodic boundaries of the voxel space, with periodic padding.

Both the autoencoder (encoder and decoder module) and the manipulation network have some uncommon features. The convolution operations use periodic padding on the x and y dimension to account for periodic boundary conditions on the voxel space. A kernel size of 3 × 3 × 3 implies that for full-size convolution we should pad each map with a border of width 1 before applying the kernel. Instead of the usual zero-padding, we fill the border with the content of the opposite edges and corners of the map. Figure 3 shows an example of a cloth extending over the edges of the voxel space, with periodic padding applied. Periodic padding is applied at every convolution and up-convolution throughout the encoder and decoder modules. All connections in the encoder and decoder are initialized with random values from the [−0.05, 0.05] range.

The manipulation network is a multi-layer perceptron with a number of modifications. One modification is the introduction of an aspect of Residual Learning (He et al., 2015). As can be seen in Figure 2, the network is comprised of three vertical sections, colored in blue, green and yellow in the figure. The blue section of the input layer receives the encoded state representation. In each subsequent layer, the blue section receives a copy of the activation vector on the blue section of the preceding layer (i.e., the pink connections in Figure 2 have fixed weights of 1.0). Activations computed in the layer are added to the copied values. The blue section essentially serves to hold the state representation as it is incrementally modified through the layers. Note that with all other weights set to 0, this architecture simply passes on the encoded state unchanged.

This style of propagation, where setting all mutable weights to 0 results not in blank output but in pass-through behavior, was originally proposed to facilitate the training of very deep networks (He et al., 2015). The result is a learning style where subsequent layers learn to make incremental improvements to the representation as it propagates through the net. Our network is not particularly deep (the manipulation module has just 10 layers), and our implementation differs (the residual connections do not skip layers), but the concept of incremental modification is applicable to our problem setting. Pre- and post-manipulation cloth states often show some degree of resemblance. A short displacement distance often leaves part of the cloth undisturbed. Many movements displace the cloth in space but leave parts of its shape intact. Hence a computation style of incremental modification seems appropriate. In the experimental results below, we include a variant without these residual connections (Configuration C1) to assess the effect of their inclusion.

Neurons in the green section behave as in a regular neural network. They serve to compute the appropriate modifications and apply them to the state representation. This section has no residual connectivity. Finally, the yellow section receives the manipulation input. The manipulation input is small (6 values) compared to the state representation in the blue section and the activation vector in the green section (512 values each). We offset this imbalance in two ways. Firstly, we initialize the weights on connections from manipulation inputs to larger values (random values from the [−0.05, 0.05] range) than the rest of the weights in the manipulation network (random values from the [−0.001, 0.001] range). Secondly, manipulation inputs are provided (identically) at every layer. This avoids the need for the network to retain the manipulation signal through numerous layers before it can affect computation in the upper layers. We included a variant that feeds the manipulation input only into the first layer of the manipulation network (configuration C0), to assess this feature's effect on performance.

At the borders between any two modules (i.e., on the output of the E and M modules), we introduce a simple discretization layer.

(4) $$activation_{out}=\;\frac{round(res\cdot activation_{in})}{res}$$

Here *res* is a system parameter controlling the grain of the discretization, which we set to 16. This layer is not differentiable, so during backpropagation we let the gradient pass through unmodified, cf. (Van den Oord et al., 2017). The use of discrete latent representations in generative models has recently been reported on in Jang et al. (2017), Maddison et al. (2017), where it is typically motivated by the latent variables' correspondence to categories. Our motivation to discretize representations is rather different (there is no concept of categories in our task). We will be using the manipulation module recurrently to compute multi-step plans, which can quickly incur a build-up of noise and diffusion in the latent representations. If we let the latent representation be discrete, it can be denoised by means of rediscretisation. Specifically, if the magnitude of the noise on a given representation falls below 0.5/*res*, then equation (4) will return the representation perfectly denoised. Theoretically, as long as the error incurred in a single pass through the manipulation network falls below this threshold, predictions would not lose accuracy as the number of passes through the network increases. Conversely, without discretization, any error larger than zero would carry through to the next pass, leading to degradation of prediction quality as the number of passes increases. Hence *in theory*, the discretization layer may improve multi-step prediction and planning ability. Of course, setting *res* too low will harm the expressiveness of the latent representation, as it reduces the number of possible latent representations. We included a non-discretizing variant in our experiments (configuration C3), to assess the effect on performance.

## Planning Algorithm

The network as discussed so far computes predictions of manipulation outcomes, but its actual purpose here is plan generation. Here we discuss how the net is used to generate multi-step manipulation plans, and how planning and manipulation execution are interleaved in operation.

### Plan Generation

**Figure 4:**
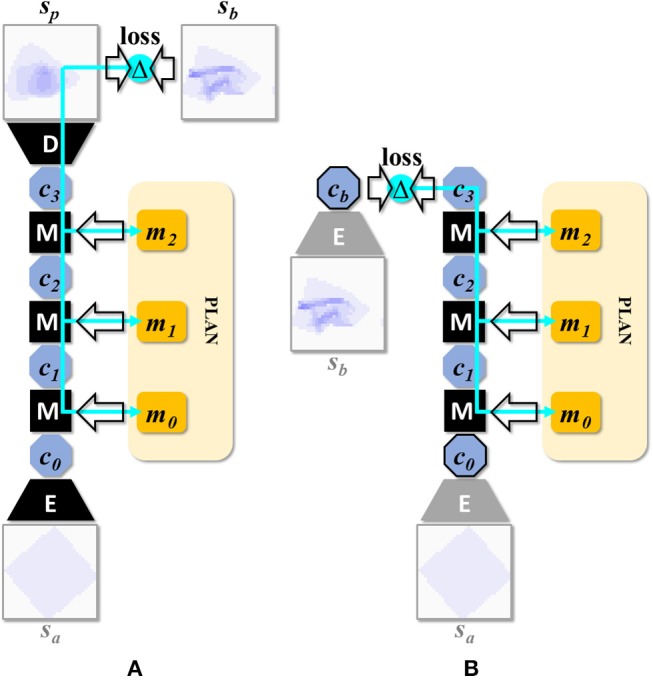
**(A)** Network setup for generating a 3-step plan. This composition can be denoted as *D(M(M(M(E(s*_0_*),m*_0_*),m*_1_*),m*_2_*))*, or EM^3^D for short. Multi-step manipulation planning is done by back-propagation through such recurrent applications of the manipulation network. The cyan arrows represent the back-propagating error signal. **(B)** Network setup after speed optimization. By precomputing latent encodings of s_a_ and s_b_, plan search can be run entirely in latent space, avoiding repeated (and comparatively expensive) propagations through the encoder and decoder modules.

By applying the manipulation network recurrently for *n* times, we can predict the outcome of an *n*-step plan. We refer to a net with *n* recurrent passes through the manipulation module as EM^n^D, and to these nets in general as EM^*^D nets. By means of iterated backpropagation w.r.t. the manipulation inputs, these nets can be used to generate multi-step manipulation plans. Figure 4A illustrates the concept for *n* = *3* (the maximum considered in the present work). This generation process can be further optimized for speed by precomputing latent representations of the start and end state and running the iterative plan generation process in latent space entirely. Figure 4B illustrates this optimization. Note that the encoder is used twice while the decoder is not used. Algorithm 1 specifies the procedure for generating a plan *m*_*ab*_ for transforming state *s*_*a*_ into state *s*_*b*_.

**Algorithm 1:**
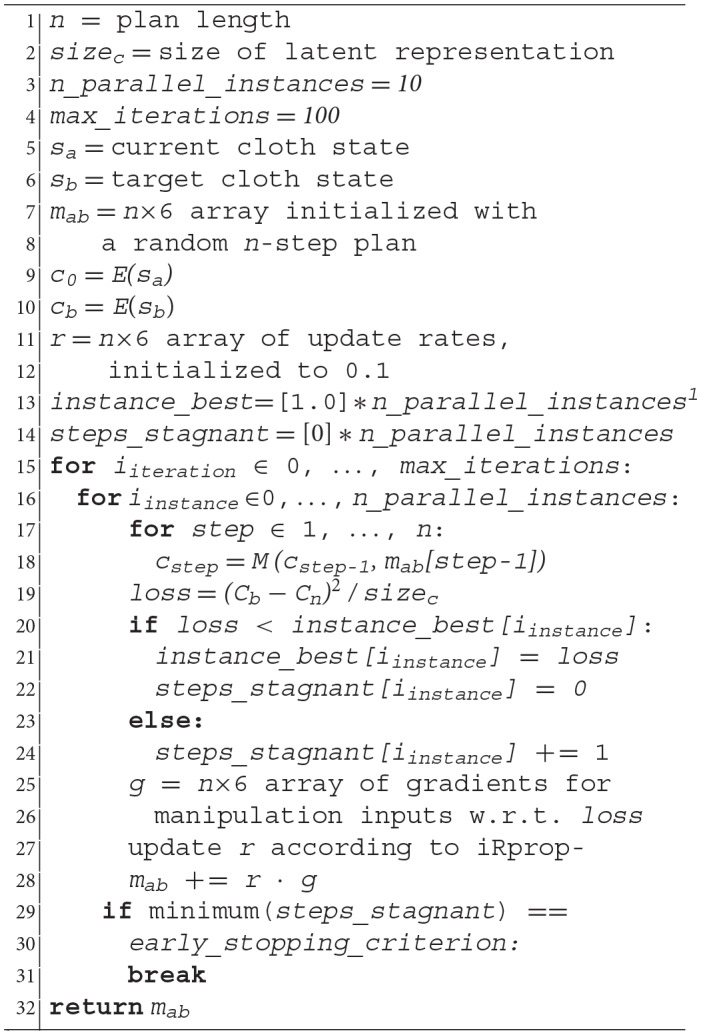
Plan generation.

Although the optimized variant has a substantial speed advantage, its viability was found to depend on the loss functions used for training, so for some experiments below we report scores for both variants. An earlier report on this work (Arnold and Yamazaki, 2017) also employed the non-optimized variant.

Manipulation input values are adjusted by means of the iRprop- variant (Igel and Hüsken, 2000) of the Rprop update scheme (Riedmiller and Braun, 1992). Rprop was proposed as an update rule for neural network training. The distinguishing feature of Rprop and its variants is that a separate learning rate η_*i*_ is kept for each variable *v*_*i*_ to be optimized. Typically, the variables are neural network connection weights, but in the present case the object of optimization is the manipulation input, so we keep one learning rate for each variable in *m*_*ab*_. The learning rate is updated every iteration of the optimization process, on basis of the sign of the error gradient at its variable. When the sign of the gradient is unchanged w.r.t. the previous iteration, the learning rate is multiplied by η^+^, and the variable is updated by *-*η times the sign of the gradient. When the sign of the gradient has flipped, different variants of the Rprop algorithm operate in subtly different ways. The iRprop- variant multiplies the learning rate by η^−^, leaves the variable's value unchanged, and blocks change of the learning rate at the subsequent iteration. Learning rates are clipped to the range [Δ_*min*_, Δ_*max*_]. Rprop and its variants are robust against a broad range of initializations of the learning rates, and can quickly zoom in on solutions, even on error functions with small gradients, as only the sign of the gradient is used. A drawback is the need for individual learning rates for each variable, but in our use case the number of variables to be optimized is small (6*n*). We found values of 1.5 for η+ and 0.33 for η*-* to perform well in our setup. Learning rates are initialized to 0.1 and the learning rate bounds Δ_*min*_, Δ_*max*_ were set to 10^−10^ and 0.1.

We set the number of search instances (*n_parallel_instances* in Algorithm 1) to 10, and let the instances run in parallel on GPU (i.e., the for loop at line 16 is parallelized). Each instance is started from a different random initialization. Search is cut short if all search instances are stagnant for a set number of iterations (*early_stopping_criterion*, set to 25 here). We observed that in practice, most runs run for the full number of iterations (*max_iterations*, set to 100 here), although improvement during the latter half of the search tends to be marginal. We adopt the plan with the lowest remaining loss value as the final result, and obtain its expected outcome state by forward propagation through EM^n^D.

### Closed-Loop Planning

Here we describe the procedure for assessing the system's planning performance, used to generate the results in the next section. We adopted a “closed-loop” procedure that interleaves planning and execution steps (Algorithm 2). The execution step here refers to performance of the first step of the generated manipulation plan. In our test setup, this means that we send the manipulation instruction to the simulator, which then executes the manipulation and returns the resulting cloth state. Interleaving planning and execution ensures that small errors do not build up over multiple manipulations, and affords some degree of correction when outcomes are not as expected. Alternatively, faster but less accurate performance can be achieved by “open-loop” operation: planning just once and performing the obtained sequence “blindly” (i.e., without observing and re-planning w.r.t. the intermediate results).

**Algorithm 2:**
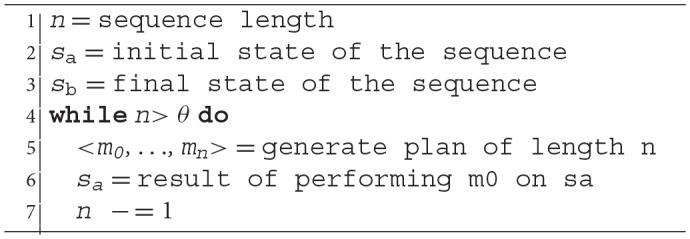
Closed-loop planning.

## Data Generation

We generate states using the cloth simulation functionality of the Blender 3D editor (Blender, 2017) (version 2.77a). The cloth is represented by an 80 × 80 mesh with the cloth modifier enabled. The mesh measures 1.4 × 1.4 in Blender's spatial units, spanning from [−0.7, −0.7, 0.03] to [0.7, 0.7, 0.03] in its initial configuration. In the conversion between Blender data and neural network input, the neural network's input space corresponds to a viewport of size 2 × 2 × 0.25 in Blender units. The cloth mesh has no explicit thickness, but we let collision detection maintain some minimal distance between vertices, as well as between vertices and the virtual desk surface (a plane at *z* = *0*), so that the cloth behaves as if it has some thickness.

Each sequence starts with the cloth laid out as a square on a flat surface (representing e.g., a table), with the axes of the cloth aligned with the x and y axes of the coordinate system. Then randomly generated manipulations are performed one by one, while we store the resulting cloth state to the dataset after each individual manipulation. Two-handed and one-handed manipulations are generated, with equal probability. Zero-handed examples (i.e., failures to manipulate) need not be generated at this stage; such examples can be generated on the fly during training from successful examples (we return to this point below). One issue that requires consideration when generating examples is the range from which to pick the manipulation coordinates. Covering the entire space the cloth can reach over 3 manipulations is inefficient, and will grow increasingly inefficient as we consider longer sequences. We constrain the range for manipulation coordinates by shifting the coordinate system along the displacement vector of each manipulation. This way the cloth always remains near the origin.

Random grasp points are found by randomly selecting cloth vertices, and values for the displacement vector are randomly picked from the [−1.4, 1.4] range. To manipulate the cloth, we pin the vertex or vertices selected as grasp point(s) to an invisible actuator object (an “empty” in Blender terminology), and assign the relevant movement trajectory to this actuator.

**Figure 5:**
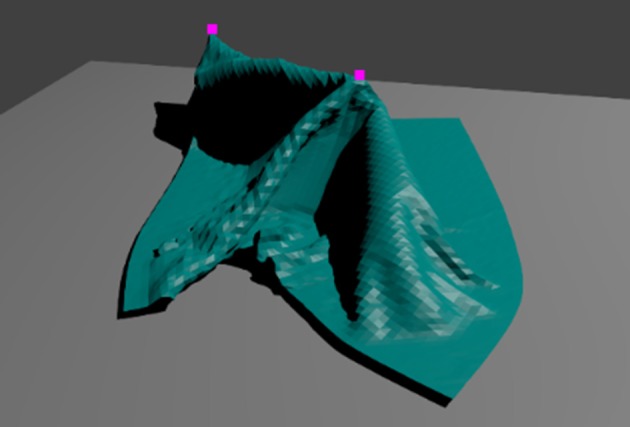
Snapshot from a cloth manipulation in progress in simulation. The pink squares show the points where the cloth is pinned to the (invisible) actuators. Cloth mesh resolution is 80 × 80.

During manipulation, movement speed of the actuators is fixed to 0.02 Blender units per frame in horizontal and vertical direction (independently). The lifting height is set to 0.15 Blender units. Horizontal and vertical actuator movement starts simultaneously. Vertical movement is stopped once the lifting height is reached. A snapshot of a manipulation in progress is shown in Figure 5. Once the movement is completed, the actuator releases the cloth, and the simulation is left to run for 16 additional frames to allow the cloth to fall down and settle.

We generate 2,900 sequences of length 3, for a total of 8,700 manipulation examples. Sequences are stored as 7-tuples of the form <*s*_0_*, m*_0_*, s*_1_*, m*_1_*, s*_2_*, m*_2_*, s*_3_>. We designate 2,000 sequences as training data, 600 as test data, and 300 as validation data. The simulation parameters defining the cloth behavior are given in Table 2. Settings not pertaining to cloth specifically were left at their default values.

**Table 2:** Cloth simulation parameter settings.

**Material settings**	
Mass	1.0
Structural stiffness	10.0
Bending stiffness	50.0
**Damping**	
Spring damping	50.0
Air damping	0.0
Velocity damping	1.0
**Collision settings**	
Collision distance	0.015
Self-collision distance	1.0

*For interpretation of these values we refer to the Blender documentation (www.blender.org)*.

Whereas all manipulation sequences in the dataset start from the initial, fully spread state, the prediction and planning capabilities of the system are not constrained to starting from this state. In our evaluation experiments below, we assess prediction and planning abilities starting from any non-final state within the sequences.

It should be noted that this data generation procedure limits the scope of the dataset to cloth states that are accessible from the initial spread out state within a few manipulations from the manipulation repertoire under consideration. As such the dataset does not represent a uniform sampling of the space of possible cloth configurations. Uniform sampling of this space is by itself far from trivial, and we have not pursued it here. Whereas by no means exhaustive, the dataset does present a broad variety of starting states. State variation is further enriched by means of data augmentation, as explained below.

### Data Augmentation

Data augmentation is performed during training by applying random rotation (rotating all cloth mesh vertices around the origin by a random angle between 0 and 360), mirroring, and grasp point swapping. A grasp point swap changes which grasp point's coordinates go into which pair of coordinate input neurons. The order of the grasp points is immaterial, so the outcome state remains the same. The data is further augmented with failure-to-grasp examples. Training on failure-to-grasp examples is necessary, since many valid manipulation inputs have neither grasp point lying on the cloth. However, there is no need to explicitly generate such examples. In case of a failure to grasp, the cloth remains in the same state, so we can generate failure-examples simply by picking existing examples, replacing the grasp points with random points falling outside the cloth, and replacing the result states with copies of the initial states. We let every batch (16 examples) contain two such failure cases. In these examples we do not shift the coordinate system along with the displacement vector.

Rotational data augmentation in particular proved essential to make training work on the relatively small dataset used here. Without it, training quickly overfit on the training data and never achieved adequate performance on the test set.

### The Role of Simulation

Although in this paper we use explicit simulation to generate data, this simulation is not an integral part of the system as it is in simulation-based planning. Simulation data is used here because it is easy to generate, but given a similar data set of real-world data the system could be trained and used without any explicit simulation. This fact that an accurate simulation model of the object is no requirement for this system is an important feature, as it is often difficult in practice to obtain accurate simulation models of cloth items and discrepancy between model and reality can substantially degrade performance of simulation-based systems. Note that our simulated cloth is not a stand-in for the real planning subject; the simulated cloth itself is the subject.

In consideration of the costs of real-world data generation, we kept the size of the simulation dataset modest, to ensure that the system can be trained effectively on realistically generatable amounts of real-world data. A system for automated data generation on robot hardware is currently under development (Tanaka et al., 2018).

## Training

We train the network on our Blender-generated dataset. Recall that the purpose of training here is not to teach the net to plan, but to let it acquire a forward model of the cloth's dynamics w.r.t. the manipulation repertoire. Hence whereas we generated sequences of length 3, the training process uses individual manipulation examples. Each 7-tuple <*s*_0_*, m*_0_*, s*_1_*, m*_1_*, s*_2_*, m*_2_*, s*_3_> provides 3 training examples of the form <*s*_*i*_*, m*_*i*_*, s*_*i*+1_>. The net is trained on 1,250,000 batches of 16 such manipulations each. Batches are composed randomly, but with some weighing of the manipulation steps. As all sequences start from the same default state, there is less cloth shape variation over the first-of-sequence manipulations in the dataset than over the third-of-sequence manipulations. The later cloth shapes in a manipulation sequences are also taller (i.e., have more vertices with higher z-coordinates) on average, as repeated manipulation often produces shapes in which the cloth is folded over itself. To counter-balance this bias in shape variation across steps we pick the first, second and third manipulation step with probabilities of 1/7, 2/7, and 4/7, respectively.

### Loss Functions

We use two loss functions, which we denote as loss_s_ and loss_c_. Loss_s_ is the mean squared error between network output, i.e., *D(M(E(s*_0_*),m*_0_*))* and the (voxel representation of) the actual outcome, i.e., *s*_1_.

(5) $$\begin{array}{r} \begin{array}{c} loss_{s}=\;\frac{\sum {\left(D\left(M\left(E\left(s_{i}\right),m_{i}\right)\right)-s_{i+1}\right)}^{2}}{size_{s}} \end{array} \end{array}$$

Here size_s_ is the size (in voxels) of the state representation. The second loss function, loss_c_, is introduced to enforce consistency of state encoding format between the input and output layers of the manipulation module.

(6) $$\begin{array}{r} \begin{array}{c} loss_{c}=\;\frac{\sum {\left(M\left(E\left(s_{i}\right),m_{i}\right)-E\left(s_{i+1}\right)\right)}^{2}}{size_{c}} \end{array} \end{array}$$

Where size_c_ is the size of a latent representation (512 with our settings). Loss_c_ serves to enable multi-step planning. Multi-step planning involves recurrent use of the manipulation module. For recurrent application to make sense, the input and output of the manipulation module must be in the same encoding format, i.e., the latent representation of a given cloth state should not differ (much) depending on whether it is read at the input or output of the manipulation module. When the encoding format is inconsistent, looping the manipulation module's output back into its input will not produce a meaningful subsequent output. Hence, consistency of representation format between the manipulation network's input and output must be enforced explicitly. However, we do not want to impose any format in particular; finding a suitable encoding is up to the learning process.

To achieve this we compare two differently obtained encodings of *s*_1_. The first is simply *ĉ*_1_ as above, i.e., *M(E(s*_0_*),m*_0_*)*. The second is obtained by application of the encoder directly on *s*_1_, i.e. *E(s*_1_*)*. Loss_c_ quantifies encoding inconsistency as the mean squared error over these two encodings of *s*_1_. Regardless of how the encoder module encodes states, this loss will be low if the manipulation module preserves the encoding format between its input and output. A functionally similar loss term was used in Watter et al. (2015).

There is overlap in function between the two loss terms. Minimizing loss_s_ trains the net to compress states into an easily manipulable format and to apply manipulations, whereas loss_c_ trains the net to keep the encoding consistent over the course of manipulation application and to apply manipulations in this encoding.

It proved difficult to balance the losses effectively. Loss_s_ and loss_c_ derive from different representation formats, and do not necessarily decrease in tandem over the training process. Balancing them with fixed weight parameters will often have one dominate the other. We resolved this issue as follows: instead of combining the losses into a compound loss function, we compute the gradients for both losses separately, and then combine the gradients on a per-weight basis by averaging over their signs. As only the signs of the gradients are used, the resulting update rule can be considered a variant of the Manhattan update rule. With combining gradients, the update rule takes the following form:

(7) $$\begin{array}{r} \begin{array}{c} \Delta w_{i}=0.5\cdot \eta \cdot \left[sign\left(g_{i}^{s}\right)+sign\left(g_{i}^{c}\right)\right] \end{array} \end{array}$$

Where Δ*w*_*i*_ is the change in weight for connection *i*, η is the learning rate, and $g_{i}^{s}$ and $g_{i}^{c}$ are the gradients for connection *i* w.r.t. loss_s_ and loss_c_, respectively. Using this rule, weights are updated by η in the direction of the sign of the gradients when the gradients agree in sign. If the signs oppose, they cancel out, and the weight is not updated. This update rule is used on the weights of the manipulation module only. No gradients for the decoder module can be derived from loss_c_, and whereas gradients for the encoder module can be derived they may actually be harmful: with respect to the encoder, loss_c_ would favor trivial encodings that map every state to the same representation, for this maximizes encoding consistency and makes manipulation application trivial. Restricting loss_c_ to the manipulation module bars this dead-end solution. For the encoder and decoder modules we use the Manhattan update rule on the gradients derived from loss_s_ only:

(8) $$\begin{array}{r} \begin{array}{c} \Delta w_{i}=\eta \cdot sign\left(g_{i}^{s}\right) \end{array} \end{array}$$

Learning rate η is initialized to 5·10^−5^, and reduced dynamically (see Learning rate adjustment and overfitting counter-measures).

Use of the Manhattan update rule is unusual. In general, it is by no means the fastest weight update rule. However, our network proved hard to train with the more common update rules. We expect that this problem is related to the strong zero-bias in our data (zeros outnumber ones by a large margin in the voxel representations of all cloth states). Experiments with more advanced rules invariably saw the net devolve into producing all-zero outputs (a fine first approximation, but hard to escape from). We never observed this problem with the Manhattan update rule. Additionally, the Manhattan update rule affords the easy combination of dissimilar losses shown in equation (7), and circumvents the vanishing gradient problem, both without additional hyperparameters to tune.

Since the use of the Manhattan rule is atypical, we include results for a configuration using standard Stochastic Gradient Descent (SGD) (configuration C7). Loss_s_ and loss_c_ are combined by simple summing. Using a modestly high initial learning rate of 5·10^−3^, training first converges upon the all-zero solution mentioned above and temporarily stagnates there, but eventually escapes and achieves some level of prediction ability.

### Alternative Encoding Consistency Enforcement

An alternative training scheme combines loss_s_ with a loss computed over *s*_*i*_ and *D(E(s*_*i*_*))*, i.e., the typical autoencoder loss. Wahlström et al. (2015) adopt a loss term to this effect. To compare these variants, we define a reconstruction loss as follows:

(9) $$\begin{array}{r} \begin{array}{c} loss_{r}=\;\frac{\sum {\left(D\left(E\left(s_{i}\right)\right)-s_{i}\right)}^{2}+\;\sum {\left(D\left(E\left(s_{i+1}\right)\right)-s_{i+1}\right)}^{2}}{2{\cdot size}_{s}} \end{array} \end{array}$$

For convenience, we let loss_r_ combine pre- and post-manipulation states (as our training examples provide both). Our experiments below include a configuration (configuration C6) that replaces loss_c_ with loss_r_ (combined with loss_s_ in the same manner as loss_c_). This configuration, too, is trained with the Manhattan update rule.

### Learning Rate Adjustment and Overfitting Counter-Measures

To appropriately adjust the learning rate as the net trains, and to avoid overfitting, we use a validation set of 900 examples (300 sequences). Every 10,000 batches, we evaluate prediction performance (i.e., loss_s_) on the entire validation set. When validation set performance has not increased for 5 such evaluations in a row (i.e., over 50,000 batches) at the same learning rate, the learning rate is reduced by a factor 2. To avoid overfitting on the training data, we store a copy of the net whenever the validation score is improved, and perform all performance assessments below on these “validation-best” networks. This strategy can be considered a simple variant of early stopping (Morgan and Bourlard, 1990; Prechelt, 2012). This learning rate adjustment scheme was applied identically in all system configurations.

## Results—Outcome Prediction

**Figure 6:**
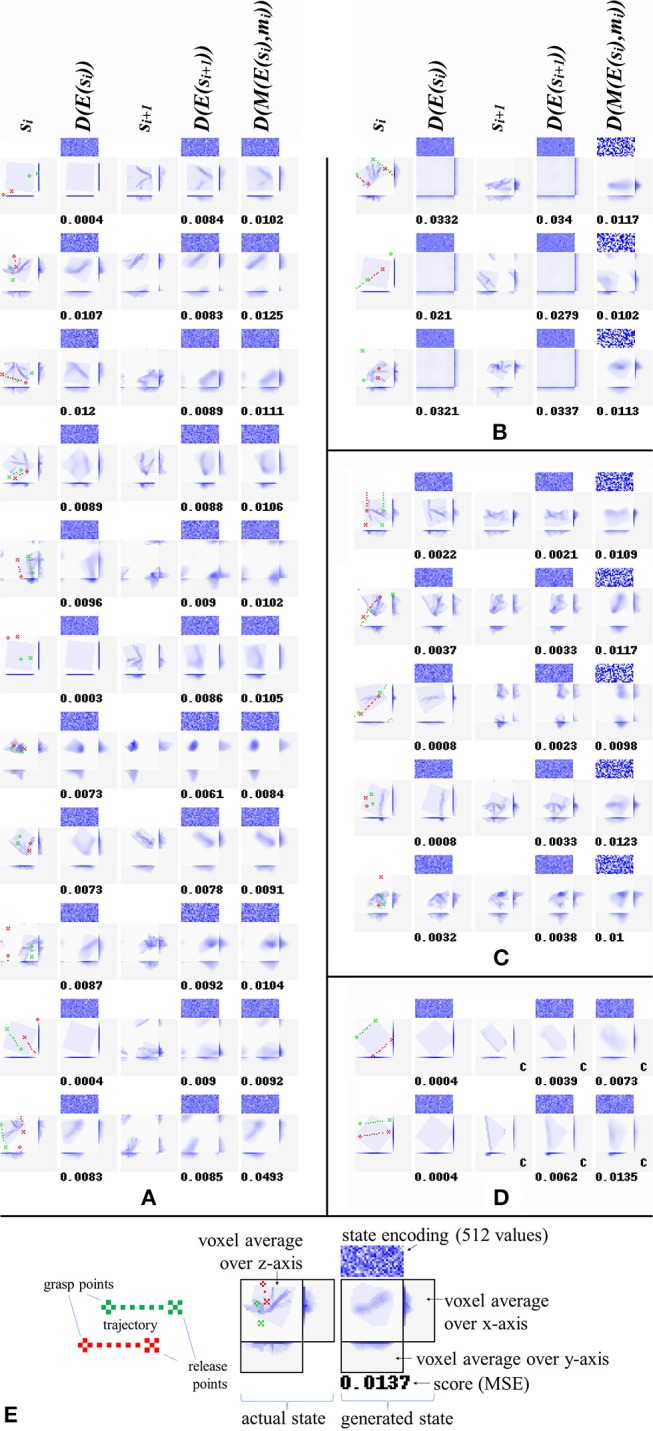
Reconstructions and predictions generated by various configurations. Each row corresponds to one result, showing (from left to right): start states *s*_*i*_, their reconstructions *D(E(s*_*i*_*))*, manipulation outcomes *s*_*i*+1_, their reconstructions *D(E(s*_*i*+1_*))*, and outcome prediction results *D(M(E(s*_*i*_*),m*_*i*_*))*. Along with each generated state is shown a direct visualization of its encoding (512 real values, shown as blue tones) and its MSE. All examples are from the test set. **(A)** Results for configuration C4. The last example in panel a shows a typical failure case (the net mistakes a grasp point right on the cloth edge for a miss, and consequently predicts no change of shape). **(B)** Results for configuration C5. Training without loss_c_ produces accurate predictions, but the “reconstructions” show no obvious resemblance to their targets. **(C)** Results for configuration C6. Training with loss_r_ instead of loss_c_ produces highly accurate reconstruction, and prediction accuracy similar to configurations C3, C4, and C5. **(D)** Prediction results for two common manipulations: folding in two along one of the cloth's axes, and folding in two along a diagonal. For ease of interpretation, we manually centered the outcomes for these examples (centered outcomes are marked with a letter *c* in their lower right corner). **(E)** Legend explaining the figure format.

Once trained, the network can fairly well predict the result of applying a given manipulation to a given cloth state. Table 3 gives results over the test (top panel) and training (bottom panel) sets for all experiments, and Figure 6 shows representative example results for configurations C4, C5, and C6. For generating these scores, all data augmentation types were enabled except for the failure-to-grasp augmentation (including this would artificially improve the scores). The binary scores are computed by rounding the values of all voxels to the nearest binary value, then taking the absolute difference w.r.t. the target state and dividing by the total number of voxels (16384).

**Table 3:** Prediction results on test and training data.

**Configuration**		**Error Measure**	***D(E(s_***i***_))***	***D(E(s_***i*+1**_))***	***D(M(E(s_***i***_),m_***i***_))***
**TEST SET**					
C0	Single input	MSE	0.00567 (0.0043)	0.00852 (0.0026)	0.0114 (0.0045)
		binary	0.00781 (0.0062)	0.0119 (0.0038)	0.0158 (0.0060)
C1	No residual connectivity	MSE	0.00724 (0.0055)	0.0107 (0.0030)	0.0120 (0.0044)
		binary	0.00990 (0.0078)	0.0148 (0.0044)	0.0165 (0.0058)
C2	Shallow M	MSE	0.00633 (0.0046)	0.00939 (0.0027)	0.0122 (0.0045)
		binary	0.00869 (0.0067)	0.0130 (0.0040)	0.0167 (0.0060)
C3	Continuous	MSE	0.00537 (0.0042)	0.00812 (0.0026)	0.0109 (0.0045)
		binary	0.00741 (0.0061)	0.0113 (0.0038)	0.0150 (0.0059)
C4	Default	MSE	0.00522 (0.0041)	0.00790 (0.0025)	**0.0107** (0.0047)
		binary	0.00722 (0.0059)	0.0110 (0.0037)	**0.0148** (0.0060)
C5	Loss_s_ only	MSE	0.0272 (0.0058)	0.0311 (0.0044)	0.0108 (0.0049)
		binary	0.0384 (0.0035)	0.0386 (0.0040)	0.0149 (0.0062)
C6	Loss_s_ & loss_r_	MSE	**0.00151** (0.0014)	**0.00245** (0.0011)	0.0110 (0.0047)
		binary	**0.00204** (0.0019)	**0.00335** (0.0015)	0.0151 (0.0060)
C7	SGD	MSE	0.0153 (0.0050)	0.0185 (0.0045)	0.0191 (0.0051)
		binary	0.0194 (0.0077)	0.0245 (0.0060)	0.0249 (0.0070)
**TRAINING SET**					
C0	Single input	MSE	0.00562 (0.0043)	0.00843 (0.0027)	0.0110 (0.0042)
		binary	0.00772 (0.0062)	0.0117 (0.0039)	0.0152 (0.0056)
C1	No residual connectivity	MSE	0.00719 (0.0055)	0.0107 (0.0032)	0.0117 (0.0041)
		binary	0.00983 (0.0078)	0.0147 (0.0046)	0.0160 (0.0055)
C2	Shallow M	MSE	0.00629 (0.0047)	0.00931 (0.0028)	0.0120 (0.0045)
		binary	0.00860 (0.0067)	0.0129 (0.0041)	0.0164 (0.0060)
C3	Continuous	MSE	0.00531 (0.0042)	0.00802 (0.0026)	0.0101 (0.0038)
		binary	0.00733 (0.0060)	0.0112 (0.0038)	0.0140 (0.0051)
C4	Default	MSE	0.00515 (0.0041)	0.00781 (0.0026)	0.00982 (0.0039)
		binary	0.00711 (0.0059)	0.0109 (0.0038)	0.0136 (0.0052)
C5	Loss_s_ only	MSE	0.0271 (0.0057)	0.0309 (0.0043)	**0.00979** (0.0039)
		binary	0.0385 (0.0036)	0.0386 (0.0040)	**0.0135** (0.0051)
C6	Loss_s_ & loss_r_	MSE	**0.00146** (0.0014)	**0.00239** (0.0011)	0.0101 (0.0039)
		binary	**0.00198** (0.0019)	**0.00327** (0.0015)	0.0138 (0.0052)
C7	SGD	MSE	0.0152 (0.0049)	0.0184 (0.0046)	0.0191 (0.0052)
		binary	0.0192 (0.0077)	0.0244 (0.0061)	0.0249 (0.0072)

*Mean squared and binary error scores, for reconstruction, D(E(s_i_)) and D(E(s_i+1_)), and outcome prediction, D(M(E(s_i_),m_i_)), for test (top panel) and training (bottom panel) datasets, under various system configurations. Standard deviations in brackets. Best scores for each item bolded*.

We also include *D(E(s*_*i*_*))* and *D(E(s*_*i*+1_*))*, i.e., the result of encoding and then directly decoding the initial state and goal state. Note however that except for configuration C6, these pathways are not trained directly. In general, the pre-manipulation state (*s*_*i*_) is reconstructed more accurately than post-manipulation state (*s*_*i*+1_). This is unsurprising: *s*_*i*_ ϵ *{s*_0_*, s*_1_*, s*_2_*}* whereas *s*_*i*+1_ ϵ *{s*_1_*, s*_2_*, s*_3_*}*, and in general states occurring later in a sequence will have more complex shapes. In particular, *s*_0_ is the same initial state in every sequence (albeit rotated by a random angle). Being simple and common, it is generally reconstructed with very high accuracy. This state never occurs in the *D(E(s*_*i*+1_*))* and *D(M(E(s*_*i*_*),m*_*i*_*)* targets.

We trained a total of 8 system configurations. Configuration C4 is the default configuration discussed so far. Configuration C0 feeds the manipulation input only at the first layer of the manipulation network, to assess the effect of feeding it anew at every layer. Configuration C1 drops the residual connectivity in the manipulation module, to assess the effect of this connectivity on performance. Configuration C2 reduces the number of layers in the manipulation module from 10 to 5, to assess whether 10 layers is overkill for this task. Configuration C3 drops the discretization layers, meaning latent representations are continuous. Configuration C5 and C6 are included to investigate the role of encoding consistency enforcement. Configuration C5 drops loss_c_, whereas configuration C6 features an alternative consistency enforcement scheme that replaces loss_c_ with loss_r_ (see section Alternative Encoding Consistency Enforcement). Lastly, configuration C7 replaces the Manhattan update rule with regular SGD as discussed above.

We compare the various configurations to our base configuration C4. Looking at the scores in Table 3 we observe that C4 outperforms C0, C1, and C2 on both test and training data, indicating that feeding the manipulation input at all layers of the manipulation network and the inclusion of residual connections is beneficial, and (at a rough granularity) that the depth of the manipulation module is warranted. C4 is also seen to outperform C3 on both sets, but the difference is marginal at best, suggesting the contribution of discretization was limited.

Despite high prediction accuracy (exceeding C4 on the training set), configuration C5 produces by far the worst direct reconstructions. High prediction accuracy despite dismal reconstruction may seem contradictory at first glance. In a standard autoencoder, low-quality reconstruction would strongly imply low-quality latent representations, and it is hard to see how accurate prediction could be achieved with low-quality latent representations. However, the low reconstruction quality observed here is no indication of poor latent representation quality, but of inconsistency of representation format between the representations produced by the encoder and the manipulation network. The decoder can only meaningfully decode representations produced by the latter, hence prediction succeeds but reconstruction fails. This result indicates that the consistency of representation format achieved by the default configuration (evidenced by the combination of high prediction accuracy and high reconstruction accuracy) is indeed due to the inclusion of loss_c_, Configuration C6 excels in reconstruction, exceeding all other configurations on both the test and training set. This is to be expected, as it is the only configuration explicitly trained to reconstruct. Overall, prediction ability is close between C3, C4, C5, and C6. Configuration C7 falls short of all other configurations in terms of prediction ability, and short of all other configurations except C5 in terms of reconstruction ability, showing that our variant of the Manhattan update rule was more effective for training this particular network architecture than standard SGD.

Looking at Figure 6A we can observe that configuration C4 produces appropriate (though moderately diffuse) reconstructions and predictions of the target states. Incidental prediction failures are observed on examples with one or both grasp points lying very close to the edge of the cloth. The cloth's edges correspond to sharp discontinuities in the relation between manipulation inputs and outcomes: grasping the cloth right at its edge produces a very different outcome from failing to grasp the cloth by a millimeter. The observed failures can often be understood as mistaking one of these situations for the other.

Figure 6B shows reconstructions and predictions from configuration C5. The “reconstructions” here bear no discernible resemblance to the targets at all. We initially thought that even in absence of loss_c_, the residual connectivity of the manipulation module may produce some degree of encoding consistency, but the outcomes do not support this notion. Nonetheless, prediction accuracy is high. Figure 6C shows reconstructions and predictions from configuration C6. Here too, prediction is close to configuration C4, whereas reconstruction is by far the most accurate across all configurations.

Figure 6D shows prediction results w.r.t. two common folds: folding in half along one of the cloth's axes, and folding in half along a diagonal. The results are centered in view for ease of interpretation. These examples were generated for purpose of illustration, and are not part of the dataset. We observe that prediction quality on these examples is in line with prediction on the random examples in the test set.

## Results—Planning

Next we assess the system's planning performance. From a manipulation sequence of length 3, represented as a 7-tuple <*s*_0_*, m*_0_*, s*_1_*, m*_1_*, s*_2_*, m*_2_*, s*_3_>, we can extract a total of six subsequences <*s*_*i*_*, m*_*i*_*, …, s*_*i*+*n*_>*: (n,i)* ϵ *[(1,0), (1,1), (1,2), (2,0), (2,1), (3,0)]*. Note that subsequences *(n,i)* for which *i* = *0* start from the fully spread state, whereas subsequences for which *i* > *0* start from states generated by application of *i* random manipulations. For *n* = *3* the only subsequence is the full sequence, and consequently planning for 3 steps always starts at the fully spread state. As in training and prediction evaluation, random rotation and mirroring is applied. For planning we apply rotation and mirroring identically to each state and manipulation in a sequence, in order to maintain sequence coherence. The other data augmentation operators are not applicable for planning.

For each sub-sequence we run the system on 100 examples, following the closed-loop planning procedure detailed in section Closed-Loop Planning. Table 4 shows the scores obtained by configurations C3, C4, C5, and C6 for both test (top panel) and training (bottom panel) datasets (C0, C1, C2, and C7 were excluded as they evidently fell short in prediction ability). Scores represent the mean absolute errors between the goal state and the state actually obtained by performing the planned manipulations in simulation, both in voxel representation (since both are binary representations, this is identical to the MSE). Medians were included because the presence of occasional failures sometimes skews the mean upward. As is to be expected, there is some falloff in accuracy as plans get longer, but recognizable approximations of the goal state are obtained for all plan lengths tested here. What performance gap there is between training and test data appears to be below the noise level of this assessment, suggesting that the net did not overfit substantially and generalizes well to unseen data.

**Table 4:** Planning results for test and training data.

**Test set**			**Sequence**					
**Configuration**			**1-0**	**1-1**	**1-2**	**2-0**	**2-1**	**3-0**
C3	Continuous	μ	0.0134	0.0166	0.0181	0.0210	0.0254	0.0243
		σ	0.0063	0.0069	0.0099	0.0066	0.011	0.0083
		M	0.0130	0.0174	0.0170	0.0210	0.0237	**0.0224**
C4	Default	μ	0.0130	**0.0153**	**0.0177**	**0.0206**	**0.0250**	**0.0236**
		σ	0.0080	0.0064	0.010	0.0063	0.0082	0.0063
		M	**0.0123**	**0.0151**	0.0163	**0.0206**	**0.0232**	0.0226
C5	Loss_s_ only	μ	0.0401	0.0439	0.0430	0.0512	0.0519	0.0557
		σ	(0.019)	(0.016)	(0.018)	0.013	0.012	0.011
		M	0.0406	0.0442	0.0438	0.0513	0.0518	0.0561
C5^*^	Loss_s_ only	μ	**0.0128**	0.0167	0.0183	0.0335	0.0374	0.0385
		σ	0.0065	0.0070	0.011	0.012	0.016	0.015
		M	0.0127	0.0162	**0.0156**	0.0312	0.0316	0.0330
C6	Loss_s_ and loss_r_	μ	0.0346	0.0403	0.0397	0.0438	0.0460	0.0468
		σ	0.018	0.016	0.017	0.015	0.012	0.013
		M	0.0364	0.0378	0.0385	0.0447	0.0437	0.0466
C6^*^	Loss_s_ and loss_r_	μ	0.0131	0.0175	0.0180	0.0244	0.0277	0.0292
		σ	0.0067	0.0095	0.010	0.0090	0.012	0.0093
		M	0.0125	0.0161	0.0168	0.0233	0.0251	0.0272
**Training set**			**Sequence**					
**Configuration**			**1-0**	**1-1**	**1-2**	**2-0**	**2-1**	**3-0**
C3	Continuous	μ	0.0135	0.0156	0.0177	**0.0212**	0.0221	**0.0238**
		σ	0.0073	0.0055	0.012	0.0068	0.0081	0.0072
		M	0.0123	0.0150	0.0158	0.0213	0.0205	**0.0229**
C4	Default	μ	0.0124	**0.0154**	0.0158	0.0217	**0.0201**	0.0248
		σ	0.0060	0.0073	0.0086	0.0078	0.0076	0.0088
		M	0.0123	0.0148	0.0143	**0.0211**	**0.0197**	0.0240
C5	Loss_s_ only	μ	0.0405	0.0466	0.0440	0.0505	0.0511	0.0535
		σ	0.017	0.015	0.018	0.014	0.014	0.012
		M	0.0412	0.0468	0.0450	0.0522	0.0528	0.0553
C5^*^	Loss_s_ only	μ	**0.0117**	0.0165	**0.0154**	0.0308	0.0328	0.0360
		σ	0.0058	0.0094	0.074	0.012	0.015	0.014
		M	**0.0107**	**0.0142**	0.0145	0.0281	0.0282	0.0311
C6	Loss_s_ and loss_r_	μ	0.0344	0.0414	0.0380	0.0432	0.0448	0.0474
		σ	0.016	0.017	0.018	0.015	0.014	0.012
		M	0.0349	0.0410	0.0384	0.0419	0.0438	0.0463
C6^*^	Loss_s_ and loss_r_	μ	0.0129	0.0163	0.0157	0.0229	0.0268	0.0288
		σ	0.0066	0.0083	0.010	0.0080	0.011	0.011
		M	0.0132	0.0144	**0.0142**	0.0225	0.0240	0.0262

Average (μ), standard deviation (σ), and median (M) of binary errors for each type of subsequence, for the test (top panel) and training (bottom panel) datasets. For subsequence types, n-i indicates a subsequence of length n, starting at manipulation i of the source sequence. Each score is an average over 100 examples from the relevant set. Example sets used for different subsequence types are non-overlapping. Best scores (per subsequence type) in bold. Results with a

* *mark in the configuration column were obtained using an alternative planning algorithm (see text)*.

**Figure 7:**
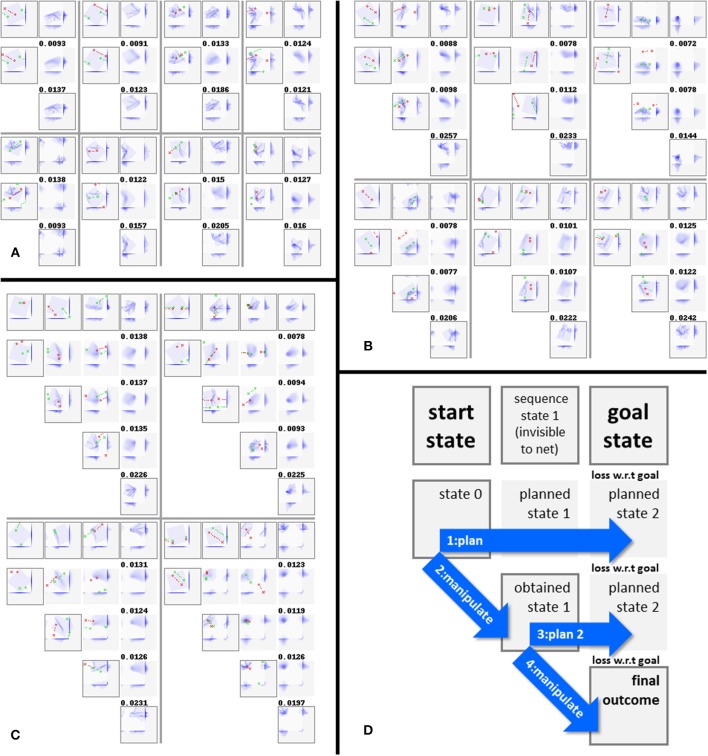
**(A–C)** Representative 1, 2, and 3-step manipulation results, respectively, for the default configuration (C4). Manipulations and intermediate states of the original sequence (top row in each example) shown for reference only; the net sees the start and goal states only. Framed states are voxelisations of actual (i.e., simulation-generated) states, non-framed states are network-generated predictions. Numbers above states indicate MSE loss w.r.t. the goal state. Note that manipulation trajectories can wrap around the edges of the viewport, and that the camera shifts along with the manipulation trajectory. **(D)** Legend explaining the figure format.

**Figure 8:**
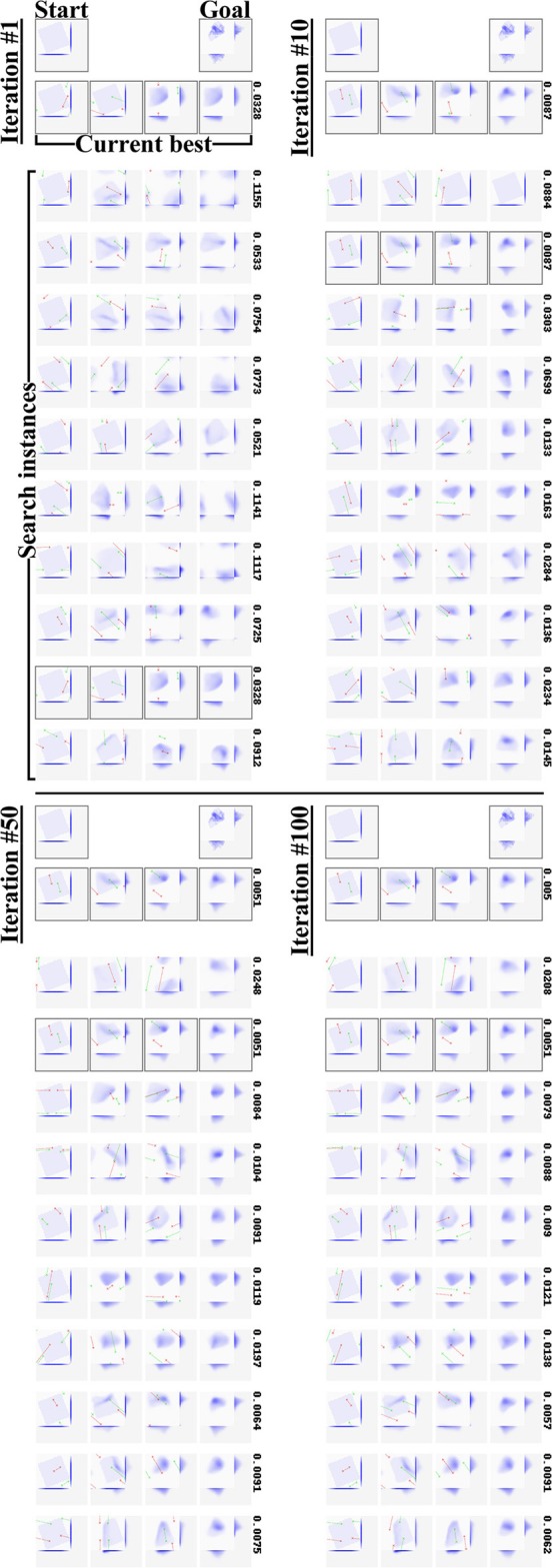
Iterations 1, 10, 50, and 100 from the generation process for a 3-step plan. Each panel shows the task, the best plan up to the iteration, and the current plan in each of the 10 parallel search instances. Numbers to the right of a plan indicate its residual error w.r.t. the goal state. We see the search instances quickly converging on a variety of plans for approximating the target outcome.

**Figure 9:**
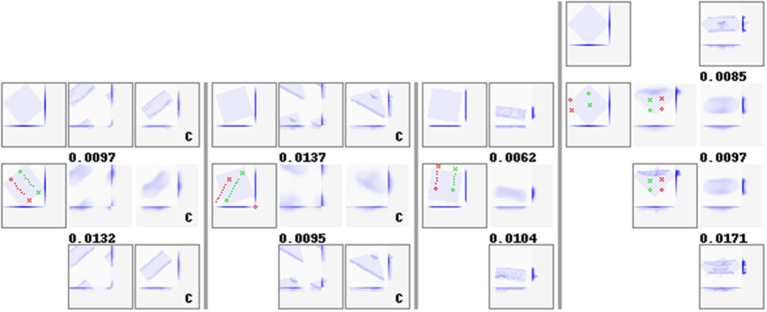
Manipulation plans and outcomes obtained for four hand-crafted goal states. See Figure 7D for the figure format. For examples that wrap around the view border (first two examples), we include manually centered views of the goal, prediction, and result states. Centered states are marked with a letter *c* in their lower right corner.

Configuration C4 succeeds in both single and multi-step planning. Figure 7 shows representative examples of plans and outcomes. Figure 8 shows a few iterations of the generation process of a 3-step plan. On a single NVIDIA GTX1080 GPU, plan generation took <2.5 s on average. Plan generation times per plan length are given in Table 5. Figure 9 shows plans and outcomes obtained for a small number of common folds. The goal states here are not part of the dataset, but were modeled manually in simulation for the purpose of illustration. We observe that the trained network is capable of generating sensible plans that produce adequate approximations of these goal states.

**Table 5:** Mean plan generation times per plan length.

	**Plan length**			
**Configuration**		**1**	**2**	**3**
C3	continuous	1.85s (0.039)	2.06s (0.047)	2.27s (0.041)
C4	default	1.90s (0.050)	2.10s (0.070)	2.30s (0.52)
C5^*^	loss_s_ only	4.33s (0.082)	4.51s (0.10)	4.65s (0.066)
C6^*^	loss_s_ and loss_r_	4.29s (0.067)	4.49s (0.083)	4.65s (0.068)

Times in seconds. All times measured over the test set. Numbers in brackets are standard deviations. Results marked with a

* *used an alternative planning scheme (see text)*.

Plan generation with configuration C5 fails when we use the algorithm given in section Planning Algorithm. However, this is in part a consequence of speed optimization (see Figure 4B). The planning process aims to reduce the loss between latent representations of the goal state and the expected outcome for the manipulation input. Comparing the two only makes sense if there is sufficient encoding consistency between the two. Configuration C5 does not enforce consistency during training, so failure here is not unexpected. To perform planning with C5, we can follow the strategy given in Figure 4A, using the loss between the voxel representations of the goal state and the expected outcome for the manipulation input. This strategy is slower (taking roughly twice as long), as we need to run the decoder every time the manipulation inputs are updated, but otherwise similarly effective. Results for C5 obtained with this alternative planning strategy are given in Table 4 as C5^*^.

Configuration C6 had encoding consistency enforced via loss_r_ and indeed fares better than C5, but still falls short of C3 and C4 by a large margin on both test and training data. Assuming that loss_r_ was not quite effective for this purpose, we ran C6 with the alternative planning scheme as well, and give the results as C6^*^.

The alternative planning scheme brings C5^*^ scores for 1-step plans up to par with C4, with C5^*^ doing slightly better on the training set and C4 doing slightly better on the test set (repeating the pattern seen for prediction). However, scores for multi-step planning remain poor. This is to be expected: without an encoding consistency enforcing loss, we obtain an M module that predicts the results of individual steps accurately, but cannot read its own output. This makes it unfit for recurrent application, leading to failure in multi-step planning.

The alternative planning scheme brings C6^*^ scores for 1-step plans close to C4 as well for both test and training data. Scores for multi-step plans are improved too, but fall short of C4. This again suggests that loss_r_ did not enforce encoding consistency as effectively as loss_c_. We hypothesize as follows: configuration C6 trains to minimize loss_s_ and loss_r_. When both are near zero, this implies that D can decode latent representation *c* of a given state *s* into an approximation of *s*, regardless whether c was produced by E or M. This suggests encoding consistency between E and M, but does not guarantee it: D may be decoding different latent representations into similar state approximations. Loss_c_ on the other hand, directly and specifically enforces encoding consistency with no such wiggling room.

Whereas C4 outperforms C3 by a slight margin on most sequences in the test set, the scores are again very close, indicating that the effect of the discretization layers was marginal at best. As seen in Table 5, plan generation with C3 was faster by a similarly diminutive margin.

It bears emphasizing why back-propagation (BP) can arrive at good solutions within 2.5 s. During plan generation, we only search for input values, not connection weights. The number of input values for a plan of length *n* is *6n*, so BP for generating a plan of a few steps has vastly fewer variables to optimize than BP in the training process has. Furthermore, planning does not work on batches of examples; only one input state and one output state need to be considered (in our implementation the batch dimension is instead used to run multiple search instances in parallel). The small scale of the problem allows us to zoom in on solutions quickly with aggressive use of iRprop-.

The plan generation process need not be implemented with BP. In preliminary experimentation, we found plan generation by means of a genetic algorithm to be viable as well (the trained network then serves as the evaluation function for solutions generated by the genetic algorithm). However, the fact that the BP machinery is already present in the system for training makes the BP approach particularly convenient to implement.

It is worth noting the planning process we employ is not deterministic. Where multiple solutions exist, different runs of the search process (and different search instances in parallel search, as seen in Figure 8) can produce different solutions.

## Self-Occlusion

So far, we have considered the case where the cloth shape is fully visible (albeit at low resolution) to the network. Full visibility would be hard to achieve in a physical implementation on robotic hardware. To be applicable in a physical setup, the system must be able to handle self-occlusion. In this section we evaluate the performance impact of self-occlusion. We assume to have a single top-down view of the cloth, recorded using a depth camera. To replicate this view limitation on simulation data, we occlude all voxels below the top-most 1-voxel in each z-column of the voxel space. Occluded voxels are given the value 1, same as known-occupied voxels. We evaluate the impact of occlusion by training the default configuration from scratch with occlusion enabled and all other settings unchanged. All states presented to the network, in training, prediction, and planning, are given with occlusion applied. Consequently, predictions are also generated with occlusion present.

Since the state representation differs from the preceding experiments, prediction results cannot be compared straightforwardly to results obtained without occlusion. However, planning performance can be evaluated as before, since it involves comparison of actual states only. As before, we compute the binary error scores over non-occluded voxel representations of the outcome and goal state. Table 6 shows the results for the occlusion experiment, for both test and training data. Comparing these results to the results obtained by configuration C4 without occlusion (Table 4), we observe that the performance impact of occlusion is small. The use of incomplete state representations carries a risk of inducing more overfitting, but we do not observe a widening of the gap between training and test scores. These results indicates that even with the occlusion incurred by a single static top-down view, the cloth state representation generally still provides sufficient information to allow effective planning.

**Table 6:** Planning results with self-occlusion.

**Occlusion**		**Sequence**					
		**1-0**	**1-1**	**1-2**	**2-0**	**2-1**	**3-0**
Test set	μ	0.0136	0.0162	0.0187	0.0210	0.0249	0.0252
	σ	0.0068	0.0073	0.010	0.0068	0.010	0.0089
	M	0.0141	0.0160	0.0173	0.0218	0.0230	0.0229
Training set	μ	0.0131	0.0165	0.0171	0.0213	0.0230	0.0236
	σ	0.0066	0.0061	0.0081	0.0062	0.0088	0.0070
	M	0.0127	0.0160	0.0164	0.0220	0.0221	0.0225

*Average (μ), standard deviation (σ), and median (M) of binary errors for each type of subsequence, for the test (top) and training (bottom) datasets. For subsequence types, n-i indicates a subsequence of length n, starting at manipulation i of the source sequence. Each score is an average over 100 examples from the relevant set. Example sets used for different subsequence types are non-overlapping*.

## Discussion

The EM*D network functions as a forward model that is differentiable, and therefor searchable, w.r.t. the manipulation repertoire it is trained on. Given an economically defined manipulation repertoire, planning is not a high-dimensional problem. In the present work, planning a manipulation sequence of length *n* is reduced to gradient descent search in a *6n*-dimensional space.

The task of multi-step cloth folding could also be cast as a model-free reinforcement learning (RL) problem, so it is worth noting the merits afforded by each approach. First off, while both approaches generate goal-directed behavior, ours generates such behavior in the form of explicit plans. There is a large conceptual difference between learning to pursue a given goal state, and learning the deformation and movement characteristics of a given task environment. This has practical consequences for the training procedure. The former requires evaluation of manipulation outputs during training (which for our task would be computationally very costly). The latter, as demonstrated here, allows training from a static database of examples. Furthermore, as this style of training is goal-agnostic, the goal state for planning can be set freely, and the planning process can be constrained using additional loss terms without having to retrain the net (which is presently proving useful in integrating the system with a physical robot platform with a limited range of motion). The cost of these merits is that the planning process is slow (taking seconds) compared to the action-generation time cost of model-free RL systems.

We found that the system emphasizes overall shape (what we might call the 3D silhouette of the cloth) over the details of how that shape is realized. For example, given as target a cloth folded neatly in two, the system will produce a plan that produces the same rectangular shape, but not necessarily with the fold on the same side. This is to be expected (as the loss used in plan generation quantifies the difference between voxel representations), but not ideal. Marking or patterning of the cloth (e.g., adding an additional color channel to distinguish the cloth's hem) can likely improve this issue, but would also constrain the applicability of the system to cloths adhering to the marking scheme.

A related issue is that predictions are rather diffuse. Some degree of diffusion is theoretically appropriate. The voxelisation introduces some ambiguity with respect to the cloth mesh. Predictions from a network trained to perfection would be distributions over the manipulation outcomes for all the mesh configurations that would produce the given input voxel representation. This diffusion could be reduced (though not eliminated) by simply using higher resolution voxel representations, at the cost of slower training and operation. However, there are also some unwanted sources of blur affecting the results presented here. One is that autoencoders tend to replicate low-frequency features better than high-frequency features. This is a well-known problem (Snell et al., 2015). Low-frequency features generally have a bigger impact on the loss function, causing them to dominate the training process. However, the fact that the reconstruction results of configuration C6 show only limited diffusion suggests that the root cause of the blur in our prediction results is not just due to an emphasis on low-frequency features. Further supporting a different root cause, we experimented with 3D SSIM (Snell et al., 2015) loss functions in hopes of improving prediction clarity, but did not obtain notable improvements over the MSE loss used in the experiments here. Adversarial training (Goodfellow et al., 2014) has been shown capable of improving the quality of generated images. It is imaginable that sharper predictions could be obtained with an adversarial approach, but it is not a given that this would contribute to better multi-step planning. Sharpness only contributes to planning performance insofar it reflects increased prediction accuracy, whereas the sharpness obtained by adversarial training is obtained by the objective of reducing distinguishability between real and generated states. Also, introducing a discriminator net would substantially complicate the system. A third factor is the somewhat stochastic nature of the data itself, caused by limitations of the cloth simulation. The simulated cloth generally does not stabilize entirely. After the grasp is released and the cloth has settled into a folded state, it continues to jitter slightly, which slowly changes the cloth shape (left to run for a long time, this jittering can even cause the cloth to unfold entirely). Our data generation procedure stops the simulation 16 frames after the grasp is released, which includes variable amounts of such jitter. This introduces a source of noise, which is likely reflected in the predictions. Adoption of a more stable cloth simulation algorithm should resolve this issue. However, the system is developed with the aim of operating on real cloth, which introduces a different set of noise sources, so exact predictions will likely remain hard to obtain.

Perhaps somewhat counter-intuitively, we see that manipulation outcomes often resemble the goal state more closely than their predictions do. Evidently the planning process is robust to some level of blur. This can be understood as follows. What is required for planning to function is that the prediction's match w.r.t. the goal state should improve (i.e., planning loss should decrease) as the manipulation input approaches the correct action. Differently put, prediction ability does not need to produce the goal state, it only needs to provide predictions that are good enough to identify the goal state among other possible outcomes. This permits a fair level of noise and blur.

Next, we position our approach among related work in generative models, control, and planning. The EM^*^D net could be considered a relative of transforming autoencoders (Hinton et al., 2011) and variational autoencoders (Kingma and Welling, 2014; Rezende et al., 2014). Whereas typical autoencoders are mainly used for compression and feature extraction, transforming and variational autoencoders are used to generate novel outputs. Dosovitskiy et al. (2014) introduced techniques to make individual values in an autoencoder's latent encoding control specific features of the representation, and demonstrated how this can be used to change specific features (e.g., color, view angle) in a controlled manner. The EM^*^D net, too, compresses and modifies representations in a controlled manner, although not by changing specific features or view angles but by applying specific manipulations.

As noted in the introduction, the use of neural networks as forward models can also be found in research on model-based control. Wahlström et al. (2015), Watter et al. (2015) similarly use neural networks to map states into a latent space, in which a variety of control problems is then solved. Both studies map high-dimensional states (images) into latent space using encoder networks, and then solve control problems in latent space. A first point to note is that whereas both these studies solve control tasks, we focused on a planning task. This is fundamentally a difference in the size of the time-step: our step units are full manipulations (representing large jumps in state-space), whereas the control studies consider smaller and more granular transitions. For convenience, we will use the term “actions” here to refer to the object of inference (i.e., control signals in the control studies, and manipulation plans in the present work). Both (Wahlström et al., 2015) and (Watter et al., 2015) employ high-dimensional observations (images) of low-dimensional tasks (2D in the former, 2D to 6D in the latter). Both systems successfully reduce these observations back to low dimensionality. However, neither compresses state representations beyond the original task dimensionality. The present work employs high-dimensional observations of an intrinsically high-dimensional task. The actual state dimensionality is, strictly speaking, 19200D (x, y and z coordinates for 80 × 80 cloth vertices). Observations are 16384D voxel representations, which the encoder compresses into 512D latent representations. The encoder's task here is not to recover or approach the task's actual dimensionality; the actual dimensionality is too large to plan on effectively. Rather, the encoder must learn a manifold of low-dimensional representations of a high-dimensional state space, that also allows for easy manipulability. Our work shows that the concept of action inference in latent space can be applied effectively under the demanding conditions of intrinsically high-dimensional real-world problems.

In terms of action inference (Watter et al., 2015) stay close to the variational auto-encoder paradigm. Transitions are performed by means of linear transformations in latent space. This is effective for small time-steps in low-dimensional control problems and has advantages in terms of solution search, but it is not clear how well imposed linearity would play in inherently high-dimensional problems with large time-steps, such as treated here. Our approach is close to Wahlström et al. (2015), combining non-linear latent state transformations with backpropagation-based solution search. The concept of planning by means of back-propagation has also been discussed and demonstrated in Henaff et al. (2017), with the purpose of extending this approach to discrete state and action spaces. A shallow recurrent neural network architecture was used there, and no mapping to latent spaces was employed. In a wider scope, planning by means of back-propagation is a special case of planning by means of gradient descent, which has a history outside the context of neural networks. The present work shows that this concept is effective on moderately deep neural architectures and in combination with manifold learning.

Sergeant et al. (2015) propose an interesting autoencoder-like architecture for control of a mobile robot which generates control signals along with a reconstruction of the robot's sensor input (laser range scan measurements). Sensor input is associated with control signals using a mix of supervised and unsupervised learning. This approach, too, targets control, and is not applicable for planning as-is, as it does not accommodate variable goal states.

Another closely related work is (Finn et al., 2016). This work combined autoencoders with RL to accomplish a variety of robotic manipulation tasks, including some on deformable objects. Here too the role of the autoencoder is to extract compact representations suitable for driving control, but in contrast to other work, the autoencoder's architecture is designed specifically to extract feature points that indicate the locations of objects in the scene. The cost function used in the RL process bears notable resemblance to our loss_c_: the encoder is applied to obtain the latent representation of the goal state, which can then be compared to the latent representation of the current state to compute the cost. However, the use of RL requires that the goal state be set at training time. Consequently the trained system does not accommodate goal variability.

Koganti et al. (2017) also employ automatically acquired low-dimensional latent representations of cloth states, but in contrast to the autoencoder-based architectures above a Bayesian Gaussian Process Latent Variable Model (BGPLVM) is used. After training on motion capture and depth sensor data, the latent variable model is used to map noisy and high-dimensional depth sensor readings to cloth configurations in a task-specific, low-dimensional manifold. This approach was demonstrated on a dressing assistance task. This example illustrates the effectiveness of aggressive dimensionality reduction and manifold learning for cloth manipulation.

Finally, Erickson et al. (2018) presents a conceptually close example of Model Predictive Control (MPC) applied in cloth manipulation (a dressing assistance task). The forward model employed here operates in the haptic domain instead of the visuospatial domain, predicting the forces an action would exert (indirectly, through the clothing item) on the subject being dressed. No mapping to a latent space is performed (as cloth shape is not explicitly represented high dimensionality is less of a hurdle), and the task is again one of control rather than planning, but the approach is close to ours in its use of a recurrent neural network architecture for prediction, and its goal-agnostic training procedure. Like in our approach, the latter allows for goal definition at run-time, and hence the approach can in principle accommodate goal variability without retraining.

When training a forward model with the intent to use it recurrently, encoding consistency is crucial. Our results demonstrate that training with a loss computed over the encoding-prediction pathway (loss_c_) results in better planning ability than training with a loss computed over the encoding-decoding pathway (loss_r_).

Our network architecture is specialized for the purpose of modeling state transformations. It consists of a section of neurons designated to hold the state representation (passed on via residual connections), a section of regular neurons, and repeated action input at every layer. Our results demonstrate that this architecture is beneficial for modeling cloth's forward dynamics in latent space. As the reasoning behind this architecture is not specific to cloth manipulation, its benefits potentially extend to other task domains as well, although this remains to be investigated.

With the eye on future practical application, it is important to consider how goal state representations could be set. Although we have not focused on this aspect yet, we can outline a few ways forward. A cloth item already in the goal state could be used to specify the goal state. This would be practical when folding a number of items when at least one similar item already in the intended goal configuration can be observed. Targets could also be acquired by having a human user produce the goal state once and storing it for later recall. Either approach could build up a database of goal states on the side for quick selection of a suitable goal state at a later time (either by the user or by a high-level planning process).

Higher flexibility could be obtained by relaxing the definition of a “goal state.” There is no need for the goal state to be a literal cloth state. The planning process tries to maximize the similarity between the expected outcome and the goal. Preliminary experiments suggest that it is possible to set the goal as (a voxel representation of) the space we want to fit a given cloth item into. This allows us to consider scenarios like the following: A household robot is tasked with tidying up a room. A high-level planning process decides to store some scattered cloth items in a closet drawer. The drawer's internal sizes are estimated and passed to the cloth manipulation planning system for use as goal state. The manipulation planning system then returns a suitable manipulation sequence, allowing the robot to achieve its high-level goal.

## Future Work

A limitation of the current system is that we need to set the length of the sequence to plan, which in practice will generally be unknown. Along with the manipulation plan, the system outputs the plan's expected loss w.r.t. the target state, providing a natural quality assessment of the plan. This can be used to automatically search for the appropriate sequence length sequentially. Alternatively, it may be possible to search over variable plan lengths simultaneously. One could use an appropriately defined loss over the sequence of latent representations generated by the propagation loops through the manipulation module (with a small penalty term for plan length). Implementation and evaluation of such procedures remains as future work.

Related to the above, dynamic plan length adjustment during operation could be exploited for failure recovery. Discrepancy between predicted and observed outcome can be used to infer failure. For example, in the case of a failure to grasp, one will want to add one step in order to allow a retry of the failed manipulation.

Another avenue for improvement is expansion of the manipulation repertoire. In particular, the present manipulation format enforces that both grasp points are moved by the same displacement vector. This restriction should be relaxed, as there are common manipulations in manual cloth folding that involve different movement vectors for different grasping points. However, allowing diverging trajectories for the grasp points also introduces manipulations into the repertoire that would pull the cloth apart, so this expansion requires some careful consideration.

Concurrent to further development of the planning system, we are integrating the system with a dual-armed robot. Initial results are reported in Tanaka et al. (2018). The system as discussed in the present paper assumes certain idealizations that do not carry over to real-world application. One difference to account for is the assumption of an infinite desk and point-sized non-colliding actuators with infinite range of motion. The constraints imposed by a finite desk and real robot hands can be accounted for with constraints on the planning process in the form of additional loss terms, but work remains in defining these loss terms efficiently and balancing them with the main planning loss.

## Conclusions

We proposed the EM^*^D neural network architecture for generating multi-step cloth manipulation plans, and experimentally demonstrated its viability on simulated cloth. This approach to manipulation planning combines flexibility (variable start and goal states) speed (plans are generated in seconds), and robustness to cloth self-occlusion, core prerequisites for practical application in household robotics. Future work will focus on accuracy improvement, expansion of the manipulation repertoire, and continued integration with robotic hardware.

## Author Contributions

SA and KY contributed conception and design of the study and contributed to manuscript revision and approved the submitted version. SA implemented the system, performed the experiments, and drafted the paper.

### Conflict of Interest Statement

The authors declare that the research was conducted in the absence of any commercial or financial relationships that could be construed as a potential conflict of interest.
